# Presumptive self-diagnosis of malaria and other febrile illnesses in Sierra Leone

**DOI:** 10.11604/pamj.2013.15.34.2291

**Published:** 2013-05-26

**Authors:** Rashid Ansumana, Kathryn H Jacobsen, Aiah Albert Gbakima, Mary Hamer Hodges, Joseph Morrison Lamin, Tomasz Andrzej Leski, Anthony Peter Malanoski, Baochuan Lin, Moses John Bockarie, David Andrew Stenger

**Affiliations:** 1 Centre for Neglected Tropical Diseases, Liverpool School of Tropical Medicine, University of Liverpool, UK; 2 Mercy Hospital Research Laboratory, Bo, Sierra Leone; 3 Njala University, Bo, Sierra Leone; 4 Department of Global & Community Health, George Mason University, Fairfax, Virginia, USA; 5 Metabiota Inc., Freetown, Sierra Leone; 6 Centre for Bio/Molecular Science and Engineering, U.S. Naval Research Laboratory, Washington, DC, USA

**Keywords:** Malaria, fevers, self-care, health services accessibility, community pharmacy services, West Africa

## Abstract

**Introduction:**

The objective of this study was to evaluate the prevalence of self-diagnosis of malaria and other febrile illnesses in Bo, Sierra Leone.

**Methods:**

All households in two neighboring sections of Bo were invited to participate in a cross-sectional survey.

**Results:**

A total of 882 households (an 85% participation rate) that were home to 5410 individuals participated in the study. Of the 910 individuals reported to have had what the household considered to be malaria in the past month, only 41% were diagnosed by a healthcare professional or a laboratory test. Of the 1402 individuals reported to have had any type of febrile illness within the past six months, only 34% had sought a clinical or laboratory diagnosis. Self-diagnosis of influenza, yellow fever, typhoid, and pneumonia was also common.

**Conclusion:**

Self-diagnosis and presumptive treatment with antimalarial drugs and other antibiotic medications that are readily available without a prescription may compromise health outcomes for febrile adults and children.

## Introduction

The overuse or inappropriate use of antimalarial medications and antibiotics is a growing concern in many parts of the world, including Sierra Leone, a post-conflict country in West Africa. In Sierra Leone, these drugs are dispensed by government-run clinics and hospitals, private healthcare facilities, licensed pharmacies, and through the informal sector [1]. Because medications are readily available without a prescription, people with self-diagnosed infections can access treatments without first seeking a formal clinical consultation and/or laboratory-confirmed diagnosis. A survey conducted in April 2009 in several parts of Sierra Leone — including the city of Bo, the focus of this paper — found that 50.8% of anti-malarial drugs were dispensed without a prescription [1].

Easy access to antimalarial drugs and other antibiotic agents may compromise patient health outcomes. If the medications purchased for an infection are inappropriate — such as antibiotics taken for a viral infection or chloroquine taken for a chloroquine-resistant case of malaria — or if medications are not taken in the appropriate dosage and for an appropriate length of time, self-diagnosis and treatment may contribute to prolonged illness, more severe morbidity, and an increased risk of mortality. A study in Bo district in 2008 found that only 48.3% of malaria-positive patients at a hospital that offered free care completed the full course of prescribed antimalarial treatment [2]. Given that low rate of adherence to prescribed treatment regimens, it is likely that few patients who access over-the-counter antibiotics complete a full course of an appropriate, government-recommended formulation. The antimalarial medications available over-the-counter are rarely the formulations recommended by the national government and the World Health Organization (perhaps a good sign that national health system drugs are not leaking into the private market), and the 2009 study found a very low level of knowledge about national and international antimalarial policies and regulations among private-sector sellers of medication, compared to a high level of knowledge among public-sector providers [1].

Furthermore, misuse of antimicrobial agents can contribute to the emergence of drug resistance, which is a growing concern in Sierra Leone. A 2002-2003 study found treatment failure among more than half of pediatric malaria patients treated with chloroquine, and treatment failure was also frequently observed for sulphadoxine-pyrimethamine (SP) [3]. These results suggest that drug-resistant malaria is already common in the study area. Evidence of drug-resistant bacterial infections has also been reported, including outbreaks of multidrug-resistant *Shigella dysenteriae* in 1999-2000 [4] and drug resistant *Staphylococcus aureus* among children during a 2008-2009 study in Freetown [5]. Sierra Leone has one of the highest rates of multidrug-resistant tuberculosis (MDR-TB) in sub-Saharan Africa [6].

Over-diagnosis of malaria, in particular, may be common. Even clinicians can find it difficult to diagnose malaria accurately solely based on symptoms. A study in the Bo district in 2005 found that 82% of children suspected by clinical officers to have malaria based on physical symptoms such as fever, splenomegaly, and vomiting tested positive for parasitemia by a Paracheck-pf® rapid diagnostic test (RDT) and 18% did not [7]. Between 2004 and 2006 in the same region of Sierra Leone, only 65% of the Paracheck® RDTs performed on pediatric patients with clinically — suspected malaria were positive, suggesting that antimalarial medication may have been significantly over-prescribed without the use of confirmatory laboratory tests [8]. It is important to note that some children in highly-endemic areas test negative for malaria at the beginning of a febrile illness, such as a case of pneumonia, but then become malaria-positive later in their course of illness due to their weakened state. (Additionally, many children test positive for malaria even when they are asymptomatic.) Parents without clinical training might be more likely than healthcare professionals to diagnose any febrile illness as malaria and to seek presumptive antimalarial treatment. While presumptive treatment may be helpful — and perhaps even lifesaving — when the child actually does have malaria, a misdiagnosis may result in delayed treatment for the actual cause of the fever, and the delay in seeking professional medical care may increase morbidity and mortality [9]. The goal of this paper was to evaluate the prevalence of self-diagnosis of malaria and other febrile illnesses in Bo, Sierra Leone's second largest city.

## Methods

**Sampling Strategy.** All households within the Kulanda Town and Njai Town sections (neighborhoods) of the city of Bo were eligible for participation. The research laboratory has previously mapped all of the buildings within these sections and conducted a household census, in 2010, to identify which structures were residential ones [10]. The resulting geographic information system (GIS) was used to create a map of all of the homes within the study community. In June 2012, members of the research team visited each household on the map, as well as 11 new households (which were added to an updated map), to ask for their participation. Of the 1038 households in these sections, 882 (85.0%) agreed to participate. For each of these households, one adult, usually the head of the household, was interviewed to gather information about the whole household.

**Data Collection.** Each interview began with questions about the household's environmental characteristics, such as building materials and access to utilities, and about household demographics, such as the age and sex of each current resident. Students at boarding school, adults working in another town and not sleeping at the residence in Bo, and others who spent at least 6 months of the past year living elsewhere were not considered to be current household members.

Then a series of questions were asked about febrile illnesses experienced by household members. These questions were developed in consultation with residents of the Kulanda Town / Njai Town sections to ensure clarity, and asked about all household members of all ages. First, the household representative was asked whether anyone currently living in the household had been ill with what the household considered to be malaria in the past one month. If malaria was reported to have occurred, follow-up questions asked where those with malaria were diagnosed (such as at a hospital or clinic, at home by a nurse, or at home by an untrained person — that is, self-diagnosis) and whether they were tested for malaria by a laboratory. A second set of questions asked whether anyone currently living in the household had a febrile illness earlier in 2012 (that is, in the 6 months prior to the interview). Follow-up questions asked about the frequency and duration of febrile illnesses; symptoms associated with these fevers (such as joint tenderness, headaches, altered behavior, and jaundice); and whether the febrile person was examined by a clinician and/or had a laboratory test to determine the cause of the fever. A final set of yes/no questions asked whether the household considered anyone currently living in the household to have ever had any of more than a dozen listed communicable and non-communicable conditions, and whether those diagnoses were made by a doctor or were self-diagnosed.

**Data Management and Analysis.** Responses were entered by the interviewers directly into a Filemaker Pro 12 relational database on a password-protected tablet computer. Households were identified by a number linked to a map stored at the research laboratory; these codes were random and not related to the geographic coordinates of the map. Data were analyzed using the statistical software program SPSS (version 20). Proportions, means, and standard deviations were used to describe the variables. Chi-squared tests were used to compare rates in independent populations, such as different age groups.

**Ethical Considerations.** Adults ages 18 and older were interviewed after providing informed consent, which was documented with a signature or a thumbprint. The consent form and study materials were available in both English and Krio, the local language in Sierra Leone. No compensation or other incentive was offered. To protect the confidentiality of information shared with the research team, no names or addresses were entered into the database. The data entered into each tablet computer were deleted daily after the data files on the tablets were downloaded to a password-protected desktop computer in a locked and guarded research facility. The research protocol was approved by the Sierra Leone Ethics and Scientific Review Committee and by Njala University (Sierra Leone), the Liverpool School of Tropical Medicine (UK), the U.S. Naval Research Laboratory (USA), and George Mason University (USA).

## Results

The 882 participating households contained 5410 individuals, with a mean household size of 6.1 persons. The households reported a somewhat diverse set of socio-environmental characteristics. While 1186 (66.7%) of the 1778 beds reported to be located in the participating homes were said to have bednets, 328 (37.2%) of households reported having no bednets. In total, 877 (99.4%) of households reported seeing a rat in the house in the past month, 860 (97.5%) reported seeing cockroaches in the house in the past month, 677 (76.8%) had a tile or concrete rather than dirt floor, 546 (61.9%) had a trash bin in the home (of which 153 covered the bin), 353 (40.0%) had electricity, and 155 (17.6%) had a drinking water source within 50 meters of the home.

In total, 675 (76.5%) of the 882 households reported at least one case of malaria (as defined by the household) in the month prior to the survey, with a total of 910 (16.8%) of the 5410 individuals reported to have had malaria during that time period. However, 540 (59.3%) of these 910 individuals were presumptively diagnosed by the ill person or a household member, and only 370 (40.7%) were diagnosed following laboratory testing.

A total of 1402 (25.9%) of the individuals within participating households were reported to have had any type of febrile illness (whether caused by malaria or another condition) within the past six months. The rate of fever reported differed by age group and by sex, with young children (those 0 to 4 years old) having the highest rate (p<0.001) and females reporting more fevers than males (p<0.001) (Table 1). Only 33.9% of people with fever were reported to have had laboratory tests to determine the cause of the fever. There were significant differences in the likelihood of testing by age (p=0.011). Households with indicators of higher socioeconomic status (SES), such as those with electricity in the home or a drinking water source very near to the home, generally reported slightly lower rates of febrile illness within the household (Table 2). Markers of household SES were not significantly associated with reported testing rates.

**Table 1:** Prevalence of reported febrile illnesses and testing in the past 6 months, by age and sex

Age Group	Total			Females			Males			p-value for Chi-squared test of difference by sex (2-tailed)	
	n	n (%) reported to have had fever	Of those reported to have had a fever, n (%) who had tests to determine the cause of the fever	n	n (%) reported to have had fever	Of those reported to have had fever, n (%) tested	n	n (%) reported to have had fever	Of those reported to have had fever, n (%) tested	reported fever cases	testing for cause among those reported to have had fever
0-4	705	368 (52.2%)	136 (37.0%)	348	193 (55.5%)	72 (37.3%)	357	175 (49.0%)	64 (36.6%)	0.088	0.885
5-14	1527	423 (27.7%)	128 (30.3%)	835	233 (27.9%)	77 (33.0%)	692	190 (27.5%)	51 (26.8%)	0.847	0.169
15-29	1739	293 (16.8%)	99 (33.8%)	973	181 (18.6%)	53 (29.3%)	766	112 (14.6%)	46 (41.1%)	0.027	0.040
30-44	803	136 (16.9%)	58 (42.6%)	423	86 (20.3%)	30 (34.9%)	380	50 (13.2%)	28 (56.0%)	0.003	0.018
45-59	381	95 (24.9%)	36 (37.9%)	180	56 (31.1%)	19 (33.9%)	201	39 (19.4%)	17 (43.6%)	0.009	0.351
≥60	255	87 (34.1%)	19 (21.8%)	134	52 (38.8%)	12 (23.1%)	121	35 (28.9%)	7 (20.0%)	0.090	0.749
Total	5410	1402 (25.9%)	476 (34.0%)	2893	801 (27.7%)	263 (32.8%)	2517	601 (23.9%)	213 (35.4%)	0.001	0.309

1402 (25.9%) of household members reported fever, of whom 476 (34.0%) were tested to determine the cause of the fever.  In total, 801 (27.7%) females and 601 (23.9%) males reported fever, and 263 (32.8%) of females and 213 (35.4%) of males with fever were tested.  The rate of fevers reported was different by sex and by age group, but there were not significant age and sex differences in testing.

**Table 2:** Household environmental characteristics and reported febrile illnesses in the household (HH)

Household (HH) feature (among 882 households)	1+ HH member reported to have had malaria in the past month			Of those HHs with malaria, 1+ person with malaria reported to have had malaria testing			1+ HH member reported to have had any febrile illness in the past 6 months			Of those HHs reporting fever, 1+ person with fever reported to have had a formal diagnosis		
	among HHs with this feature	among HHs without this feature	p-value	among HHs with this feature	among HHs without this feature	p-value	among HHs with this feature	among HHs without this feature	p-value	among HHs with this feature	among HHs without this feature	p-value
Fewer than six individuals in the household (n=552, 62.8% of HHs)	417 (75.3%)	258 (78.7%)	0.252	125 (30.0%)	102 (39.5%)	0.011	453 (81.8%)	269 (82.0%)	0.932	129 (28.5%)	172 (63.9%)	<0.001
Households with at least one bednet (n=691, 78.3%)	517 (74.8%)	158 (82.7%)	0.020	176 (34.0%)	51 (32.3%)	0.686	584 (84.5%)	138 (72.3%)	<0.001	248 (42.5%)	53 (38.4%)	0.387
Having a tile or concrete floor rather than a dirt floor (n=677, 76.8%)	508 (75.0%)	167 (81.5%)	0.055	177 (34.8%)	46 (27.5%)	0.081	539 (79.6%)	183 (89.3%)	0.001	217 (40.3%)	84 (45.9%)	0.183
Having a trash bin in the home (n=546, 61.9%)	388 (71.1%)	287 (85.4%)	<0.001	138 (35.6%)	85 (29.6%)	0.105	428 (78.4%)	294 (87.5%)	0.001	163 (38.1%)	138 (46.9%)	0.018
Having electricity (n=353, 40.0%)	264 (74.8%)	411 (77.7%)	0.320	90 (34.1%)	133 (32.4%)	0.641	271 (76.8%)	451 (85.3%)	0.002	112 (41.3%)	189 (41.9%)	0.880
Using a drinking water source within 50 meters of the home (n=155, 17.6%)	106 (68.4%)	569 (78.3%)	0.010	27 (25.5%)	196 (34.4%)	0.070	124 (80.0%)	598 (82.3%)	0.505	52 (41.9%)	249 (41.6%)	0.949

Malaria and febrile illnesses were significantly more likely to be reported by households without at least one bednet, those with a dirt floor, those without a trash bin in the home, those without electricity, and those without a drinking water source near the home. Testing of at least one household member was less likely to be reported by smaller households and those without a trash bin in the home

Self-diagnosis of several other conditions was common (Table 3). All of the 234 individuals reported to have ever had influenza, as defined by the household, indicated a self-diagnosis. More than 96% of the 160 persons reported to have had what the household considered to be yellow fever (which had been the focus of a recent vaccination campaign [11]) reported self-diagnosis, as did more than 60% of the 445 persons reported to have had typhoid (which is a relatively common laboratory diagnosis in Bo, as per Widal tests). More than half of the 317 people reported to have had what the household designated as pneumonia were self-diagnosed. However, diagnosis of less common and more specific conditions, such as bacterial meningitis, hepatitis B, and hepatitis C, were nearly always reported to have been diagnosed by a doctor and not self-diagnosed by the household.

**Table 3:** Reports of family medical history, by source of diagnosis

Condition	n (%) of households reporting that someone currently living in the household has *ever had this condition*	Of households reporting this condition, n (%) reporting that the condition was *diagnosed by a doctor*	Of households reporting this condition, n (%) reporting that the condition was *self-diagnosed*
Influenza	234 (26.5%)	0 (0%)	234 (100%)
Lassa fever	5 (0.6%)	0 (0.0%)	5 (100%)
Yellow fever	160 (18.1%)	6 (3.8%)	154 (96.3%)
Common cold	774 (87.8%)	180 (23.3%)	594 (76.7%)
Asthma	79 (9.0%)	21 (26.6%)	58 (73.4%)
Typhoid fever	445 (50.5%)	177 (39.8%)	268 (60.2%)
Pneumonia	317 (35.9%)	151 (47.6%)	166 (52.4%)
Bacterial meningitis	10 (1.1%)	8 (80.0%)	2 (20.0%)
Hepatitis B	5 (0.6%)	5 (100%)	0 (0.0%)
Hepatitis C	1 (0.1%)	1 (100%)	0 (0.0%)
Tuberculosis	0 (0.0%)	0 (0.0%)	0 (0.0%)
Rift Valley fever	0 (0.0%)	0 (0.0%)	0 (0.0%)
Dengue fever	0 (0.0%)	0 (0.0%)	0 (0.0%)

Households reported a high rate of self-diagnosis for conditions such as influenza (100% of reported cases self-diagnosed), yellow fever (96.3% self-diagnosed), colds (76.7% self-diagnosed), typhoid fever (60.2% self-diagnosed), and pneumonia (52.4% self-diagnosed).

## Discussion

We found that the majority of febrile illnesses in Bo, Southern Province, Sierra Leone, are self-diagnosed without clinical examination or laboratory testing, including the more than half of suspected malaria cases that are treated presumptively without any clinical diagnostics. The fact that households with greater numbers of individuals were more likely to report that at least one household member had been tested for a febrile illness supports the validity of reports about diagnosis and testing rates, since a greater number of persons in the household increases the probability of a severe illness occurring for at least one resident. The validity of the survey instrument is also supported by the low numbers of households reporting uncommon diseases such as bacterial meningitis unless these conditions were diagnosed by a doctor.

These results are similar to those from studies in other parts of West Africa which have found that more than half of adults self-diagnose fevers and self-medicate for what they consider to be malaria [12, 13]. Of those who self-treat, only a small proportion know the correct dosage for common antimalarial medications [12].

This high rate of self-treatment is concerning, since it is likely that a significant proportion of these presumptive cases are treated inappropriately (as per the introduction to this paper). Some people who would benefit from antibiotic and supportive therapy may not be receiving adequate care, and many people who purchase medication for their self-diagnosed malaria or other conditions may be taking drugs that are ineffective for their condition. Febrile individuals who self-treat may experience disease complications and increased treatment costs resulting from delayed access to appropriate medications and other therapy [14]. Taking the wrong medication, such as taking antimalarials for a fever caused by a different infectious agent, generally means that the actual infection remains untreated for at least several days.

When tests are available at a reasonable cost, testing before treating may significantly reduce the inappropriate use of antimicrobial medications. For example, a cohort study of children ages 1 to 10 years in Uganda found that only 32% of fevers were caused by malaria, and the researchers concluded that a test-before-treating approach in that study population would reduce the use of antiparasitic drugs by two-thirds [15]. The reduction of unnecessary treatment achieved by a test-then-treat approach will, by definition, be lower in higher-endemicity areas, including parts of Sierra Leone, where a recent community-based study in the Bo area found that 83% of febrile women and young children who were tested had malaria [16]. Even so, testing may significantly reduce the number of people taking unnecessary medications. The 17% of the 17,130 tested individuals who were malaria-negative in the Bo area study represented nearly 3000 people who avoided the unnecessary use of antimalarial drugs [16].

Testing does not have to occur in a clinical setting. In rural Sierra Leone, free malaria testing by community health workers (CHWs) has increased access to diagnosis for thousands of households [16]. Those who test positive for malaria are treated by the CHWs, and those with negative malaria tests or complicated malaria cases are referred to a nearby hospital for advanced care (although the follow-up rate for referrals from this program has been shown to be very low). In some places, testing and treatment by CHWs has been found to be preferred over home treatment [17]. However, one potential challenge to testing programs is convincing healthcare providers and patients not to prescribe or take antimicrobials after a negative test result. A study from Ghana found that more than half of patients who tested negative for malaria were prescribed antimalarials anyway [18], and some studies from other parts of Africa have found similar results [19]. Taking antimalarial medication “just in case” may be seen as the best option when households do not have the resources to travel to and pay for further clinical examination and testing.

Besides the direct benefits to patients, expanded use of testing before treatment may slow the further development of drug resistance by reducing the proportion of the population accessing pharmaceutical agents without prescriptions tailored to their actual diagnoses. Patients who have seen a clinician and have a confirmed diagnosis may be more likely than others to complete a full course of an appropriate antibiotic or antimalarial, especially if their treating clinicians counsel them about the importance of compliance with prescribed treatments (an occurrence dependent on those practitioners having the time and resources to provide health education). Community-based behavior change communication processes may also help to promote healthy use of pharmaceuticals by households and communities, but when these public health structures are not in place the burden of health education typically falls on clinicians.

**Limitations.** This study had several limitations that require a conservative interpretation of the findings. No laboratory tests were conducted to confirm the reported causative agents for participants’ febrile illnesses, so we do not know how often their self-diagnoses were accurate. Additionally, participants were not asked about their use of antibiotic and antiparasitic drugs, such as what medications they preferred to take when febrile or where they procured these medications. Because these questions were not part of the survey, we have limited information about whether the self-treatment used by febrile participants was appropriate for their illnesses.

## Conclusion

This study provides evidence that self-diagnosis and self-medication for malaria and other febrile illnesses is common in Bo, Sierra Leone. In order to better understand the implications of self-diagnosis and presumptive treatment on patient health outcomes, we recommend that further studies evaluate the types of infections common in this population to see how well clinical laboratory results match self-diagnoses. Future research should also examine the pharmaceutical access and use habits of local residents to see whether appropriate courses of medication are being taken by those with and without prescriptions from a clinician. Understanding the knowledge, attitudes and beliefs, and health practices and behaviors of residents regarding diagnosis and treatment of fevers may contribute to improved health services, policies, and practices.
